# The Fascinating World of Forensic Sciences: Multidisciplinary Approaches in Human Remains and the Role of Forensic Pathology in Calabrian Experience

**DOI:** 10.7759/cureus.62209

**Published:** 2024-06-12

**Authors:** Saverio Gualtieri, Matteo Antonio Sacco, Francesco Maria Galassi, Elena Varotto, Alessandro Pasquale Tarallo, Maria Cristina Verrina, Lucia Tarda, Pietrantonio Ricci, Isabella Aquila

**Affiliations:** 1 Medical and Surgical Sciences, Magna Graecia University, Catanzaro, ITA; 2 Department of Anthropology, Faculty of Biology and Environmental Protection, University of Lodz, Lodz, POL; 3 Archaeology, College of Humanities, Arts and Social Sciences, Flinders University, Adelaide, AUS; 4 Medical and Surgical Sciences, Institute of Legal Medicine, Magna Graecia University, Catanzaro, ITA

**Keywords:** post-mortem interval, putrefaction, death, anthropology, forensic sciences

## Abstract

Forensic sciences play a vital role in the criminal justice system by providing insights into the identity of victims and suspects, causes of death, and other crucial pieces of evidence. In this research paper, we will explore the utility of forensic sciences, its techniques and applications, and the critical role of the forensic pathologist in analyzing human remains. For this purpose, we have analyzed a series of human remains and cadavers found in a state of decomposition, illustrating the medico-legal investigations carried out. Specifically, 50 cases from Calabrian experience are reported from 2003 to 2023 in different contexts of both judicial and archaeological interest and discovered by chance. In any case, anthropological, odontological, genetic, entomological, and forensic radiological investigations were carried out with the supervision of the forensic pathologist in all cases. The varied composition of the sample made it possible to understand the methods and the various specialists involved in such diversified cases. Furthermore, a review of the scientific literature on the topic of human remains was carried out. In particular, by delving into these topics, we aim to provide a comprehensive understanding of forensic anthropology and forensic sciences and their significance in the criminal justice system.

## Introduction

Forensic investigations of human remains require a combination of expertise from different branches of forensic science. Forensic anthropology plays a crucial role in the identification of human remains, particularly in cases where the remains are extensively decomposed or skeletonized [1]. Forensic anthropologists work in a multidisciplinary approach along with law enforcement and medical science specialists, including ballistics, explosives, pathology, serology, and toxicology, to determine the sex, age, and unique features of a decedent, document trauma to the body, and estimate how long a corpse has been decomposing [2-4]. Many of the indicators commonly used in identification, such as fingerprints and postmortem iris recognition, can be rapidly destroyed during the process of decomposition [1]. Therefore, forensic anthropologists are often called upon to provide valuable information about the identification of human remains. Anthropological activities such as search and recovery, determining species, age at death, sex estimation, stature, postmortem interval (PMI), and ancestry are crucial to narrowing down the search for missing persons [1]. They also evaluate trauma, such as blunt and sharp force trauma or gunshot wounds.

In addition to traditional identification methods such as fingerprinting and dentition analysis, newer techniques like isotope analysis have proved to be promising for human identification in the last decade. Forensic archeology is a relatively new field that uses archeological methods to exhume crime scenes, including bodies. Forensic archeologists work in concert with other forensic experts in DNA analysis, physical matching, forensic entomology, and forensic odontology to examine evidence. The shape of pelvic bones is the best evidence for determining the sex of the person. Typical activities, diet, and particular lifestyles are reflected in bones, while the stages of growth and development in bones and teeth can determine whether remains represent a child or an adult. Forensic technology, including 3D facial reconstruction software, can also be used to investigate human remains, although it requires specialized training and education. Overall, forensic sciences have a wide range of real-world applications in investigating human remains.

In order to facilitate timely identification, it is important to have a multidisciplinary team that includes first responders and experts working together for the management and identification of the dead in large disasters. With the help of these advanced techniques and a collaborative approach, forensic sciences can assist in identifying victims of natural disasters, enabling survivors to be eliminated from the missing persons list and providing closure for families.

In this work, we describe a case study about the discovery of human remains in the Calabrian territory. In particular, we illustrate the medico-legal investigations that have been performed demonstrating the importance of the forensic pathologist and the questions to be addressed in solving cases relating to human remains.

## Materials and methods

Case series analysis

In this study, we proceeded with the analysis of a series of cases found at the Institute of Forensic Medicine of the Magna Graecia University of Catanzaro. The selected cases included the discovery of human remains or unidentified subjects. In any case, we highlight that an inspection was carried out in the area where the remains were discovered. For each case, a cataloging of the remains was carried out and an external examination of the corpses on the scene with a photographic survey. After, in each case, an external examination of the remains found was then repeated in the morgue, evaluating the injuries that were measured and photographed. Furthermore, in cases where only bones were present, they were subjected to cleaning procedures for the purposes of a more detailed analysis.

A full autopsy was subsequently performed for non-skeletal human remains. In these cases, the operators proceeded to open the head, chest, and abdomen with evaluation and section of all the organs, if still preserved. Subsequently, tissue samples were taken and stored in formalin. For the cases subjected to an autopsy, the histopathological investigation was carried out with embedding of the samples in paraffin and staining with hematoxylin and eosin. Skeletonized human remains were measured and an anthropological study was requested with the execution of anthropometric measurements aimed at understanding sex, age, and the presence of any injuries.

In all cases where larvae were found, an entomological investigation was required to trace the time of death and any other information on the movements of the remains. The larvae found were kept in alcohol and subjected to macroscopic and microscopic evaluation and photographic measurements. In particular, entomological investigations were carried out on the marine fauna found in the deaths in water to evaluate the PMSI (postmortem submersion interval).

In cases where there were identification problems, forensic odontological consultancy was requested to investigate the age of the subject. Measurements were carried out with a radiographic examination of the dental arches. In cases where it was not possible to identify the subject due to loss of anatomical features, genetic consultancy for DNA extraction was requested. The genetic investigation was requested in cases with issues of personal identification. We used PCR extraction and amplification techniques to analyze samples of bone (femur), teeth, or body fluids if they were present. These results were subsequently given to the Judicial Authority for comparison with cases and genetic profiles of missing subjects.

In cases where the backdating of the remains was uncertain, an in-depth investigation was carried out with the radioisotope Carbon 14. The aim of this investigation was to backdate the age of the remains found in order to understand whether they could be cases of judicial interest or historical interest.

In particular, in one case of exhumation, during the COVID-19 emergency, a microbiological investigation was carried out for the detection of the SARS-CoV-2 virus. A product investigation was carried out on the corpses in which clothing was still recognizable. Furthermore, the RISC form (for unidentified corpses) was filled in for each cadaver of the judicial group.

Review of the literature

In the second phase, a literature review was performed using the PubMed NCBI search engine, Google Scholar, and Scopus. The search words have been entered: human remains and forensic sciences, forensic anthropology, forensic pathology and human remains, and genetic and human remains. The texts were examined by two researchers separately. All the titles and abstracts were read and the papers most relevant to the researched topic were selected through an operator-dependent evaluation. Works that were not in the English language and papers that were not related to forensic science or forensic anthropology investigations were excluded. In particular, only cases of interest that fell within our inclusion criteria were selected for reading the full paper.

## Results

**Figure 1:**
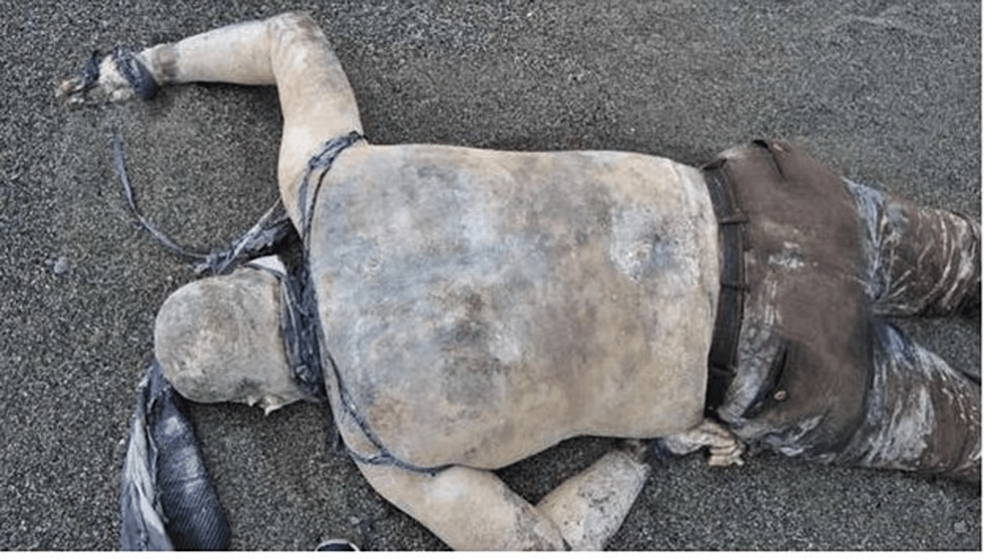
Cadaver found on the sea

**Figure 2:**
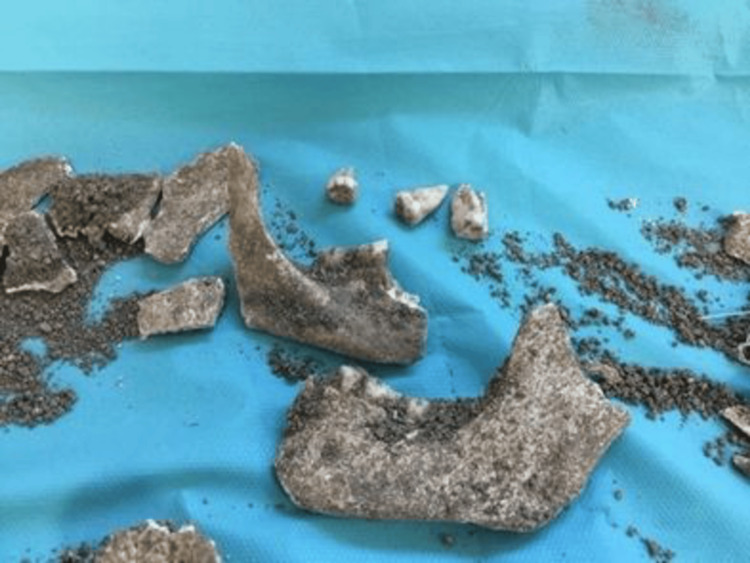
Analysis of brown bones

**Figure 3:**
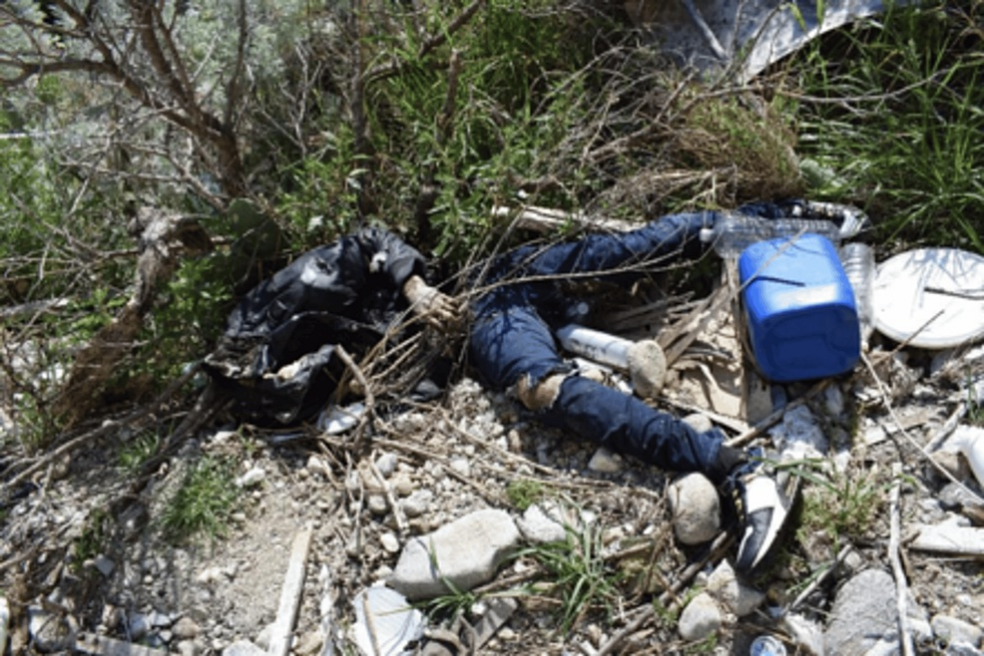
Cadaver with different putrefactive phases

The Calabrian region has the peculiarity of being a place with different geographical areas at different altitudes and a varied composition of the territory. It consists of coasts and mountain ranges with large hilly areas. The forensic pathologist who finds himself operating in such diversified contexts must take into consideration the peculiarities of this very varied region and implement different forensic methods also on the basis of the geographical area or the context (outdoors or indoors - on land or in water). Fifty cases have been analyzed in the last 10 years (Figures 1-3). 

These 50 cases were divided into two groups, namely, judicial cases and non-judicial cases. Among the cases of judicial features (40 cases), human remains from exhumations of corpses were analyzed for reasons of in-depth analysis of the cause of death and clarification of the method of death; exhumations of corpses dating back even 10-20 years analyzed in-depth for personal identification, the cause, and method of death; remains found in an aquatic environment or on the beach for reasons of clandestine landings and/or for other causes; murders with dismemberment; suicides with a late discovery of the remains; accidental deaths with the discovery of the bodies weeks after the incident; and human remains found in the water or on the beach due to clandestine landings or died due to other methods. Among the non-judicial cases (10 cases), human remains dating back to the era of Magna Graecia resurfaced for construction work, and exhumations of ancient burials in mountainous areas were analyzed. In all cases collaboration with forensic anthropologists was essential. 

## Discussion

The cases described show the importance of forensic pathologists and medico-legal investigations in solving cases concerning human remains or those in an advanced state of decomposition, in which numerous difficulties can emerge due to the postmortem transformative processes and the need to respond to the authority's questions with scientific methodology. The questions that concern the discovery of human remains are inherent to the time of death (with the aim of evaluating whether the case is of historical or judicial interest), the cause of death, the manner of death, personal identification, and comparison with other missing subjects. In the case series examined, all cases were solved and the forensic pathologist made use of the collaboration of various specialists following a multidisciplinary approach including the forensic anthropologist, the forensic odontologist, the forensic radiologist, the entomologist, and the geneticist.

Forensic anthropology is a discipline that uses methods of physical anthropology to assist law enforcement in the investigation of crimes and other legal cases. These include sex and age estimation methods to identify individuals as well as techniques to identify trauma and taphonomic markers [5,6]. In particular, in our cases reported, these forensic activities were carried out for human remains found outdoors that were partially or totally decomposed or skeletonized [7]. In our cases, we have used the methods of modern forensic anthropology for these purposes. The cases were subjected to anthropological examination, which made it possible to provide information on the sex and age of the subjects.

Forensic anthropologists use various techniques, and, in particular, sex estimation is an essential step of the analysis [8]. In the context of the analysis of human remains in forensic science, the forensic pathologist plays a crucial and demanding role. In these cases, the forensic pathologist is primarily responsible for identifying victims beyond recognition, and they are also at the helm of a multidisciplinary team of experts in a disaster situation [9]. Furthermore, they also play a role in determining the cause and manner of death and may attend death scenes as part of the investigation process [10]. In the field of forensic science used for the evaluation of human remains, the forensic entomologist is a valuable adjunct to the pathologist and is responsible for determining the period of insect activity based on the composition of the arthropod community found on the cadaver or the age of developing immature insects and testing in the court [10]. In some cases reported in the groups described above, the forensic pathologist was assisted by the entomologist, especially, in the decomposed corpses found in the open environment. This assessment allowed the time of death to be identified more precisely.

The literature review carried out showed that forensic anthropology can be beneficial in criminal investigations in a variety of ways. It can help determine the time since death and provide important evidence in criminal investigations involving human remains [11,12]. Many of the cases studied and reported by us were in an advanced stage of decomposition. In recent years, there has been a growing interest in decomposition-related studies, with research focusing on gross morphological changes, intrinsic and extrinsic influences, and PMI estimation. In cases of human remains, a big problem is identification. Between methods and techniques that are used to identify human remains, postmortem iris recognition can be used to identify individuals up to 34 days after death, depending on the environment [13]. Additionally, the process of decomposition is known to destroy many of the biological indicators commonly used in identification, such as fingerprints, which can survive up to four days in hot temperatures and over 50 days in cold environments [13]. To establish identification, investigators require information on the individual's age at death, sex, stature, ancestry, and PMI. In order to obtain this information, a variety of anatomical features must be assessed and compared with antemortem information, usually revealed through radiology [13]. Additionally, personal identification can be achieved through comparative analysis of fingerprints, DNA, dentition evidence, and radiological images [14]. Furthermore, isotope analysis is used increasingly to provide investigative leads for longstanding unidentified remains cases. In regards to radiographic evaluation, general trabecular bone patterns, bony contours, anomalies, and radiodensities can be compared to antemortem data to provide meaningful information for identification [14,15]. 

## Conclusions

In the cases analyzed, a multidisciplinary approach was, therefore, fundamental where the forensic pathologist and the other experts in the other forensic branches worked together, through the application of scientific methods with different characteristics but each with an identical common denominator, that is, the search for the truth and the answer to specific questions in the field of human remains that are gender, age at death, era of human remains, race, cause of death, manner of death, personal identification, geographical location of origin and permanence of the remains, PMI (postmortem interval), and PMSI in aquatic environment.

This cooperation often allows us to add results in terms of identification and comparison in the field of missing persons and better understand the causes and modalities of death in complicated cases in which putrefaction and postmortem modifications have generated forensic problems in solving cases. In other cases, this multidisciplinary method allows us to understand the antiquity of some human remains and give the right historical importance to human remains without historical recognition as in the cases reported by us by placing them in the correct historical era excluding legal competencies.
